# How Dengue Virus Circumvents Innate Immunity

**DOI:** 10.3389/fimmu.2018.02860

**Published:** 2018-12-04

**Authors:** Yu-Ting Kao, Michael M. C. Lai, Chia-Yi Yu

**Affiliations:** ^1^National Institute of Infectious Diseases and Vaccinology, National Health Research Institutes, Tainan, Taiwan; ^2^Research Center for Emerging Viruses, China Medical University Hospital, Taichung, Taiwan; ^3^Institute of Molecular Biology, Academia Sinica, Taipei, Taiwan

**Keywords:** dengue virus, interferon, RLR–MAVS, cGAS–STING, mitochondrial dynamics

## Abstract

In the battle between a virus and its host, innate immunity serves as the first line of defense protecting the host against pathogens. The antiviral actions start with the recognition of pathogen-associated molecular patterns derived from the virus, then ultimately turning on particular transcription factors to generate antiviral interferons (IFNs) or proinflammatory cytokines via fine-tuned signaling cascades. With dengue virus (DENV) infection, its viral RNA is recognized by the host RNA sensors, mainly retinoic acid inducible gene-I (RIG-I)-like receptors (RLRs) and toll-like receptors. DENV infection also activates the cyclic GMP-AMP synthase–stimulator of interferon genes (cGAS–STING)-mediated DNA-sensing pathway despite the absence of a DNA stage in the DENV lifecycle. In the last decade, DENV has been considered a weak IFN-inducing pathogen with the evidence that DENV has evolved multiple strategies antagonizing the host IFN system. DENV passively escapes from innate immunity surveillance and also actively subverts the innate immune system at multiple steps. DENV targets both RNA-triggered RLR–mitochondrial antiviral signaling protein (RLR–MAVS) and DNA-triggered cGAS–STING signaling to reduce IFN production in infected cells. It also blocks IFN action by inhibiting IFN regulatory factor- and signal transducer and activator of transcription-mediated signaling. This review explores the current understanding of how DENV escapes the control of the innate immune system by modifying viral RNA and viral protein and by post-translational modification of cellular factors. The roles of the DNA-sensing pathway in DENV infection, and how mitochondrial dynamics participates in innate immunity are also discussed.

Dengue virus (DENV) hijacks the host's cellular machinery and accesses cell resources in multiple ways to accomplish its lifecycle. Cellular immune signaling then turns on various cascades to fight back when the host cell senses this invading pathogen. Therefore, DENV confronts a series of challenges at each step of its lifecycle from virus entry to the release of mature virion. To counteract, DENV not only passively hides to escape the immune surveillance but also directly targets immune mediators to block the antiviral signaling transduction (1–3).

In this review, we discuss how the host cell activates innate immunity in response to DENV infection and the strategies DENV uses to evade the innate immune system. We illustrate the main theme of this article in Figure 1 and summarize the DENV antagonism (Table 1) described in the text.

**Figure 1:**
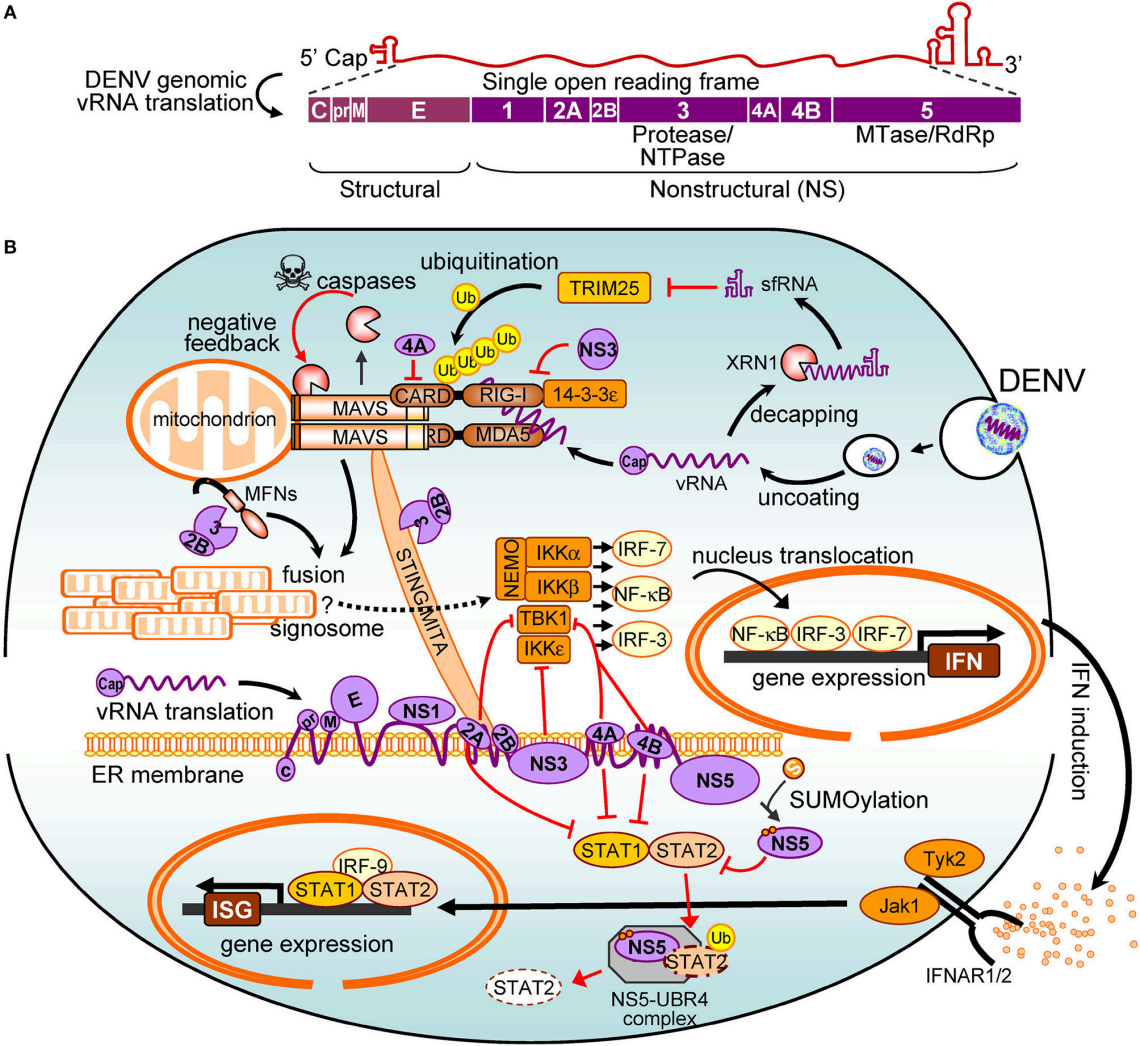
The interplay between dengue virus (DENV) and the interferon (IFN) system. **(A)** The viral proteins encoded by DENV genome are shown. **(B)** The positive signalings/pathways are illustrated with black arrows, and the antagonistic pathways are in red. Refer to the main text for details. vRNA, viral RNA; NTPase, nucleoside triphosphatases; MTase, methyltransferase; RdRp, RNA-dependent RNA polymerase; RIG-I, retinoic acid-inducible gene I; MDA5, melanoma differentiation-associated protein 5; CARD, caspase activation and recruitment domain; Ub, ubiquitin; MAVS, mitochondrial antiviral signaling protein; sfRNA, subgenomic flavivirus RNA; TRIM25, tripartite motif protein 25; MFN, mitofusin; STING, stimulator of interferon genes; MITA, mediator of IRF3 activation; NF-κB, nuclear factor kappa B; NEMO, NF-κB essential modulator; TBK1, TANK binding kinase-1; IKKα/β/ε, IκB kinase alpha/beta/epsilon; IRF, interferon regulatory factor; IFN, interferon; IFNAR, IFN-α/β receptor; STAT, signal transducer and activator of transcription; ISG, IFN-stimulated gene; Jak1, Janus kinase 1; Tyk2, tyrosine kinase 2; UBR4, ubiquitin protein ligase E3 component n-recognin 4; XRN1, 5′-3′ exoribonuclease 1.

**Table 1:** Summary of dengue virus (DENV) factors antagonizing the interferon (IFN) system.

**DENV factors**	**Target pathway**	**Actions**	**References**
sfRNA	RNA-sensing	Binds to TRIM25 to inhibit viral RNA recognition by RIG-I	(4, 5)
NS2A	IFN induction	Antagonizes the phosphorylation of TBK1 and RIG-I-induced IRF3	(6)
	IFN signaling	Inhibits IFN-triggered antiviral actions	(7)
NS2B	DNA-sensing	Targets cGAS for degradation	(8)
NS2B3	DNA-sensing	Cleaves STING through protease-dependent manner	(9, 10)
	IFN induction	Interacts with IKKε to mask part of its kinase domain to prevent the phosphorylation of IRF3	(11)
	Mitochondrial dynamics	Cleaves MFN1 and MFN2 to modulate the MFN-mediated host antiviral defense	(12)
NS3	RNA-sensing	Competes with RIG-I for 14-3-3ε binding to block RIG-I activation	(13)
NS4A	RNA-sensing	Translocates to mitochondrion-associated endoplasmic reticulum membranes to prevent the binding between RIG-I and MAVS.	(14)
	IFN induction	Blocks TBK1 activation	(6)
	IFN signaling	Inhibits of IFN-triggered gene expressions	(7)
NS4B	IFN induction	Antagonizes the phosphorylation of TBK1 and RIG-I-induced IRF3	(6)
	IFN signaling	Inhibits STAT1 phosphorylation and transcriptional activation	(7)
NS5	RNA-sensing	Catalyzes DENV genomic RNA 2'-O methylation mimicking cellular mRNA	(15)
	IFN signaling	Binds and degrades STAT2	(16–18)

*sfRNA, subgenomic flaviviral RNA; TRIM25, tripartite motif protein 25; RIG-I, retinoic acid-inducible gene-I; TBK1, TANK binding kinase-1; IRF, interferon regulatory factor; STAT, signal transducer and activator of transcription; cGAS, cyclic GMP-AMP synthase; STING, stimulator of interferon genes; IKKε, IκB kinase epsilon; MFN, mitofusin; MAVS, mitochondrial antiviral signaling protein*.

## Brief Molecular Virology of DENV

DENV belongs to the genus *Flavivirus* of *Flaviviridae* and is the leading cause of mosquito-borne viral diseases. The DENV virion harbors a messenger-sense, single-stranded RNA (ssRNA) genome that contains a 5′ cap but lacks a 3′ poly-A tail. The DENV invasion starts with cell-surface attachment and receptor binding. After internalization, the nucleocapsid is uncoated, and the virus genome then releases to the cytoplasm. The DENV RNA genome is similar to cellular mRNA, translating a polyprotein precursor in a cap-dependent manner. Viral and cellular proteases then process the polyprotein into three structural proteins (capsid [C], precursor membrane [prM], and envelope [E]) and seven non-structural (NS) proteins (NS1, NS2A, NS2B, NS3, NS4A, NS4B, and NS5). After that, viral RNA is replicated by the viral RNA-dependent RNA polymerase NS5 in the replication complex. Structural proteins are assembled with the DENV RNA genome in the endoplasmic reticulum (ER) and then transmitted to the Golgi apparatus. Ultimately, the mature and infectious virions are secreted into the extracellular space and await the next round of infection (19, 20).

DENV has evolved many strategies to minimize its exposure *in vitro* because the virus is membrane-enveloped and is liable to dysfunction *in vitro*. Thus, DENV uses the mosquito, the natural syringe, as the vector to preserve, replicate, and transmit itself. Natural feeding of human blood containing DENV viral RNA more than 5 log10-copies/ml seems sufficient to transmit all serotypes of DENV from human to the primary mosquito vector *Aedes aegypti* (21). Therefore, the period of human DENV infectiousness to the *A. aegypti* mosquitoes may vary between viral serotypes but concentrates on the days when a patient develops illness/fever (22). Despite the presence of a protein D7 capable of inhibiting DENV in mosquito saliva (23), the bites with mosquito saliva increase DENV dissemination into the mammalian host (24, 25).

DENV takes advantage of the mammalian host machinery for replication, but the immune system can detect and attack this invading pathogen. In the last decade, DENV has been considered a weak interferon (IFN)-inducing pathogen (26, 27), with the knowledge that DENV has evolved multiple strategies to antagonize the host IFN system (1–3, 28). Understanding how DENV escapes the control of innate immunity may shed some light on the complicated pathogenesis of DENV infection.

## The Concept of IFN System in Innate Immunity

Innate immunity specifies particular pattern recognition receptors (PRRs) to distinguish pathogen-associated molecular patterns (PAMPs) of invading pathogens, including both RNA and DNA viruses. The aberrant nucleic acid species in the cytoplasm, such as double-stranded RNA (dsRNA) in the endosome, cytoplasmic DNA and 5′-triphosphorylated RNA, are the unique viral PAMPs that activate corresponding PRRs (29, 30). Once activated, the sensor hands over the signal to its adaptor proteins, which then recruit kinases to phosphorylate transcription factors and ultimately turn on the production of antiviral IFNs and proinflammatory cytokines. The secreted type-I/III IFNs bind to their receptors IFNAR1/2, which activates Janus kinase (Jak)–Signal transducer and activator of transcription (STAT)-mediated signaling and leads to generation of antiviral proteins encoded in IFN-stimulated genes (ISGs) (31, 32). Various antiviral proteins interfere with steps of the viral lifecycle. For example, ribonuclease L (RNase L) is encoded by an ISG that degrades viral RNA to inhibit DENV replication (33). To counteract the host antiviral actions, DENV evolves strategies targeting various steps of the whole defense system, from sensing of the foreign DNA/RNA to the induction, signaling, and manipulation of IFN system. We categorized these various strategies by the stages of IFN system and discussed them below.

## The RNA Sensing Pathway

Camouflage is the first strategy to keep DENV away from the alarm bell of innate immunity. Similar to cellular mRNAs, DENV genomic RNA is capped at the 5′-end. Cellular mRNA is posttranscriptionally capped at the 5′-end comprising a N-7 methylguanosine and one or two 2'-O methylnucleotides (34, 35). Thus, viral RNA lacking 2′-O methylation will be recognized as non-self RNA that elicits innate immunity (35–38). DENV NS5 contains methyltransferase activity that catalyzes both N-7 and 2′-O methylations sequentially (39–41). The DENV lacking 2′-O-methyltransferase activity elicits a significant early innate immune response in host cells and thus replicates with a lower viral load than the wild-type (15). Therefore, DENV hides and stays under the radar in host cells.

DENV enters host cells by receptor-mediated endocytosis, and its RNA is released to the cytosol for translation and replication. The localization of DENV-derived dsRNA is important for recognition by PRRs. By electron tomography analysis, the cytosolic DENV dsRNA was detected in DENV-induced vesicles derived from ER membrane (42). These vesicles quarantine DENV dsRNA from the cytosolic RNA sensors in a digitonin-resistant membrane structure until 72 h postinfection (43). However, these viral RNA species, when leaked to the cytosol, also become targets for several PRRs, including melanoma differentiation-associated protein 5 (MDA5) and retinoic acid-inducible gene-I (RIG-I) in the cytoplasm and toll-like receptor (TLR)-3 in the endosome. These PRRs are essential for host defense surveillance, which synergistically recognizes DENV RNA and then initiates IFN induction (44–46). MDA5 and RIG-I are similar RNA helicases expressed in most cell types. Both contain two caspase activation and recruitment domains (CARDs) at the N-terminus for antiviral signaling initiation. After viral RNA binds to the C-terminal helicase domain, the CARD domain of RIG-I/MDA5 then interacts with the CARD domain of their downstream adaptor, mitochondrial antiviral signaling protein (MAVS) (a.k.a. IPS-1/VISA/Cardif) (47–50). This CARD–CARD interaction clusters MAVS for a signaling cascade, which is required for inducing IFN to establish an antiviral state (49). Actually, RIG-I and MDA5 recognize different RNA structures even though they share a high degree of functional and structural homology. MDA5 mainly recognizes long dsRNA or web-like RNA aggregates, whereas RIG-I preferentially senses short dsRNA and single-stranded uncapped RNA with a tri- or di-phosphate at the 5′-end (51–53). These species/forms of RNA differ from self-RNA in the cytoplasm and can be detected in DENV-infected cells (44, 54). Even though RIG-I or MDA5 alone is sufficient to potentiate DENV-induced IFN-induction signaling, RIG-I and MDA5 together trigger a higher level of IFN induction. Therefore, overexpressing RIG-I or MDA5 can suppress DENV replication; silencing of RIG-I and MDA5 contributes to DENV RNA replication and virus production (44). Because RIG-I and MDA5 share the same adaptor MAVS, lack of MAVS impairs IFN induction in DENV-infected cells (54–56).

The protein level of both RIG-I and MDA5 can be further enhanced by IFN (57), so activation of the RIG-I-MAVS pathway forms a positive feedback loop against DENV infection. In the context of RIG-I activation, the ubiquitin ligase tripartite motif protein 25 (TRIM25) binds to and adds lysine-63 (K63)-linked polyubiquitin at the CARD domain of RIG-I (58–60). The mitochondrial-targeting chaperone protein 14-3-3ε stabilizes the interaction between TRIM25 and RIG-I, thus facilitating K63-linked ubiquitination of RIG-I, which results in MAVS activation (61).

Because ubiquitination and translocation of RIG-I are both required for MAVS activation, DENV evolves strategies to antagonize this step and thus prevents RIG-I-mediated IFN responses. In DENV-infected cells, the uncapped DENV genomic RNA can be digested from the 5′- to 3′-end by the cellular exoribonuclease 1 (XRN1) leaving the incomplete degradation product subgenomic flaviviral RNA (sfRNA) (62). The DENV sfRNA binds to TRIM25, whose binding capacity depends on the sfRNA sequence, thus dampening ubiquitination-mediated RIG-I activation (4, 5). Moreover, DENV NS3 protease contains a 14-3-3ε protein-binding motif RxEP; the binding of these proteins prevents the activated RIG-I from moving from cytosol to mitochondria. Thus, infection of a recombinant DENV, with the RxEP motif replaced by KIKP, triggered a high IFN response that inhibited DENV replication (13). Also, DENV NS4A colocalizes and interacts with MAVS in mitochondrion-associated ER membranes where RIG-I relays the signal to MAVS. The TM3 domain of DENV NS4A is responsible for binding MAVS and thus prevents the association of RIG-I and MAVS (14). Therefore, DENV can disrupt the RIG-I–MAVS interaction directly to suppress IFN production.

In addition to the RIG-I–MAVS pathway, TLR3 and TLR7 are important for recognizing DENV RNA in the endosome. TLR7 senses ssRNA with G- and U-rich sequences (63), whereas TLR3 recognizes dsRNA derived from DENV replication (64). Although, both TLRs are involved in producing a type I IFN response during DENV infection, TLR3 is more effective than TLR7 in IFN induction and DENV inhibition (64). TLR7 also mediates a virus-specific humoral immune response for DENV clearance: administration of combined TLR3 and TLR7 agonists could decrease DENV replication and increase the anti-DENV humoral response in macaques (65). Even though, the direct modulation of TLRs by DENV infection remains to be seen, DENV has been shown to block TLR-mediated antiviral signaling by targeting downstream immune mediators, IκB kinase epsilon (IKKε) and TANK-binding kinase-1 (TBK1) (6, 11).

## The DNA Sensing Pathway

Despite the absence of a DNA stage in the DENV lifecycle, DENV infection still activates the DNA-sensing pathway. The cellular DNA should be located in the nucleus or mitochondria. Presence of a DNA molecule in the cytoplasm is thus expected to trigger innate immune responses, such as inflammation and IFN production (66). Cyclic GMP-AMP synthase (cGAS) is a cytosolic DNA sensor that synthesizes cGAMP, a non-canonical cyclic dinucleotide, in response to DNA stimuli (67, 68). cGAMP is a second messenger that binds and activates the adaptor protein encoded in the gene *tmem173*, namely, stimulator of IFN genes (STING) [a.k.a. mediator of IRF3 activation (MITA), or ER IFN stimulator (ERIS)] (69–71). Human STING is a transmembrane protein located on ER membrane and shares 81% similarity (68% identity) with its murine ortholog MPYS (72). After stimulation, STING is dimerized and then translocated to a perinuclear site where it forms a punctate structure and interacts with TBK1 for activating IFN regulatory factors (IRFs) (70, 71). In addition to activating IRFs and producing IFN, STING activation also triggers NF-κB signaling that leads to the production of pro-inflammatory cytokines (73, 74).

Because DENV is an RNA virus without a DNA stage in its lifecycle, the roles of the DNA-sensing pathway in DENV infection were ignored until DENV protease NS2B3 was found to cleave human STING but not its murine ortholog MPYS (9, 10). Thus, murine MPYS is more competent than human STING in suppressing DENV replication. Even though STING is not essential for IFN production stimulated by a dsRNA analog (73), STING is involved in both DNA and RNA pathogen-sensing pathways. STING can interact with RIG-I and MAVS to enhance the antiviral response (69, 70), which may suggest a crosstalk between viral RNA- and DNA-sensing pathways (75). Therefore, the possibility that DENV may target the DNA-sensing pathway to subvert innate immunity seems logical.

Stimulation of double-stranded DNA but not dsRNA analog enhances the interaction between DENV protease and STING, which then contributes to DENV protease-mediated STING cleavage (10). Therefore, the presence of DNA in cytosol upon DENV infection may contribute to DENV pathogenesis. The release of both genomic and mitochondrial DNA (mtDNA) has been proposed to activate the STING signaling pathway in DENV-infected cells (10). Indeed, aberrant DNA signal appears in cytosol and co-localizes with cGAS upon DENV infection, with the DNA signal resulting from the release of mtDNA rather than genomic DNA (8). Moreover, DENV NS2B mediates cGAS degradation dependent on autophagy–lysosome pathway to avoid IFN production (8). Even though the requirement of mtDNA in the DENV-activated cGAS–STING pathway remains unclear, the roles of mtDNA in DENV pathogenesis are of interest.

## The IFN Induction Pathway

After RNA/DNA recognition, both RIG-I–MAVS and cGAS–STING pathways recruit and activate the IKKε/TBK1 and IKKα/β/γ complexes (3). These kinases activate transcription factors, such as NF-κB and IRFs, to turn on IFN mRNA expression (29). Regardless of the multiple strategies used to antagonize RNA/DNA recognition, DENV also subverts this IFN induction step to minimize the antiviral response in infected cells (27, 76).

Although DENV protease activity is required to cleave and block STING signaling, the protease structure itself is able to inhibit IKKε kinase activity. By interacting with the N-terminus of IKKε, NS2B3 masks part of the kinase domain of IKKε to prevent the S386-phosphorylation of IRF3 (11). Despite the presence of two NS2B3-putative cleavage sites within IKKε, neither catalytic nor inactivated NS2B3 protease affects the protein level of IKKε (11). Therefore, DENV protease is able to counteract IFN induction via both catalysis-dependent and -independent mechanisms, with the wild-type DENV protease more competent than the protease-dead mutant. Moreover, DENV NS2A and NS4B regulate innate immune responses by inhibiting TBK1/IKKε-directed downstream signaling instead of targeting MAVS or STING directly (6). Thus, both NS2A and NS4B antagonize IRF3 phosphorylation resulting from the activation of RIG-I, MDA5, MAVS, TBK1,or IKKε. Only NS4A of DENV1 but not those of DENV2 or DENV4 blocks TBK1 activation (6), which suggests that DENV1 contains an additional regulatory mechanism against innate immunity.

## The IFN Signaling Pathway

DENV uses various strategies as described above to prevent the production of IFN by infected cells. Nevertheless, the secreted IFN actively binds to the heterodimeric IFN receptor, IFNAR1/2, which ultimately turns on the expression of many antiviral proteins against DENV infection. After IFN binding, the IFNAR-associated tyrosine kinases Jak1 and tyrosine kinase 2 (Tyk2) undergo autophosphorylation, which then activates downstream transcription factors, mainly STAT1 and STAT2, by phosphorylation. The phosphorylated STATs form a heterotrimeric complex with IRF9, called IFN-stimulated gene factor 3 (ISGF3), which translocates to the nucleus and awakens ISGs to fight against the virus (32, 77, 78). Meanwhile, STAT1 is also modified by the K48-linked conjugation of ubiquitins (79), which tags STAT1 for degradation and shuts off an antiviral response. Accordingly, removing these ubiquitins by the deubiquitinating enzyme USP13 increases the stability of STAT1 proteins and potentiates a stronger IFN-mediated antiviral response against DENV infection (80).

Several viral proteins of DENV are involved in suppressing the IFN-induced signaling. In the presence of IFN, the DENV NS2A, NS4A, and NS4B proteins were found to enhance the replication of an IFN-sensitive recombinant reporter Newcastle disease virus, whereas only DENV NS4A and NS4B significantly reduced the expression of a reporter gene driven by IFN-sensitive response element (7). The NS4B was found to inhibit IFN-induced STAT1 phosphorylation and nuclear translocation, which impairs the transcriptional activity of ISGF3 to turn on antiviral genes (7). Moreover, DENV NS5 can bind to and inhibit the transcription factor STAT2 activated by IFN treatment (16, 17). DENV NS5 recruits the host factor UBR4 to suppress human but not murine STAT2 via the proteasomal degradation pathway (18). Because conjugation of small ubiquitin-like modifier (SUMO) stabilizes DENV NS5 protein to maintain its biological functions, SUMOylation is required for NS5-mediated antagonism of IFN signaling (81).

## Manipulation of IFN System by Mitochondrial Dynamics

The roles of mitochondria in innate immunity were largely unknown until strong antiviral activity was detected by overexpressing the mitochondrial protein MAVS (47–50). Mitochondria move along the cytoskeleton and continuously undergo fusion and fission, which results in the diverse morphology of each mitochondrion (82, 83). MAVS forms prion-like aggregates upon activation (84), which also leads mitochondria to become aggregated in cells overexpressing MAVS (55). Therefore, manipulation of mitochondrial dynamics may regulate antiviral activity in response to virus infection. Indeed, overexpression of the mitochondrial fusion mediator mitofusin 1 (MFN1) rather than MFN2 resulted in a higher-order aggregation of mitochondria that facilitated IFN-induction signaling (12). In contrast, MAVS-mediated IFN-induction signaling was dampened in cells harboring highly fragmented mitochondrial morphology, either by overexpressing a dominant-negative MFN1 (12, 85) or by administration of a chemical disrupting mitochondrial membrane potential (MMP) (86). To manipulate mitochondria toward fragmentation, the virus may suppress fusion or enhance fission. Even though DENV infection triggers MMP disruption (55), which may result in fragmentation of mitochondria (87), the DENV protease NS2B3 alone is sufficient to cleave both MFNs and manipulate mitochondrial morphology (12). Cleavage of both MFN1 and MFN2 suppresses MFN-mediated mitochondrial fusion processes and interferes in MAVS-mediated signalings, such as IFN and cell death induction (12). Hence, mitochondria may serve as platforms transmitting the IFN-induction signal, so that aggregated mitochondria help form a more operative signosome by tethering related molecules with each other. A seemingly conflicting report showed that DENV NS4B induces mitochondria elongation and thus restricts the RIG-dependent IFN response (88). This notion is also consistent with the scenario that disrupted mitochondrial fusion or misassembled signosome leads to disturbed IFN-induction signaling in DENV-infected cells.

## Conclusions

With DENV infection, disease symptoms range from asymptomatic, classical dengue fever to life-threatening dengue hemorrhagic fever and severe dengue shock syndrome. The diverse disease symptoms result from a complicated interaction between DENV and the host. Innate immunity helps the host fight against infection by eliminating DENV and regulating follow-up immune responses. The non-canonical functions of DENV proteins and DENV-derived sfRNA in antagonizing the IFN system further damage infected cells in the battle between DENV and its host. DENV may defeat the host immunity at first line of defense. The seesaw of DENV-inducing and -antagonizing innate immunity in the initial state of infection may contribute to the DENV pathogenesis at some later time. Recent evidence shows that both viral and cellular factors are involved in the host responses upon DENV infection. Therefore, we highlight critical regulatory mechanisms of innate immunity by showing how DENV manipulates it. Notwithstanding unfinished puzzles, antiviral applications derived from all these studies are anticipated.

## Author Contributions

Y-TK and C-YY conceived and wrote the draft. Y-TK, ML, and C-YY wrote and proofread the manuscript.

### Conflict of Interest Statement

The authors declare that the research was conducted in the absence of any commercial or financial relationships that could be construed as a potential conflict of interest.

## References

[1] Morrison J Aguirre S Fernandez-Sesma A. Innate immunity evasion by dengue virus. Viruses (2012) 4:397–413. 10.3390/v4030397 22590678 PMC3347034

[2] Green AM Beatty PR Hadjilaou A Harris E. Innate immunity to dengue virus infection and subversion of antiviral responses. J Mol Biol. (2014) 426:1148–60. 10.1016/j.jmb.2013.11.023 24316047 PMC4174300

[3] Gack MU Diamond MS. Innate immune escape by dengue and West Nile viruses. Curr Opin Virol. (2016) 20:119–28. 10.1016/j.coviro.2016.09.013 27792906 PMC5578430

[4] Goertz GP Pijlman GP. Dengue non-coding RNA: TRIMmed for transmission. Cell Host Microbe (2015) 18:133–4. 10.1016/j.chom.2015.07.009 26269946

[5] Manokaran G Finol E Wang C Gunaratne J Bahl J Ong EZ. Dengue subgenomic RNA binds TRIM25 to inhibit interferon expression for epidemiological fitness. Science (2015) 350:217–21. 10.1126/science.aab3369 26138103 PMC4824004

[6] Dalrymple NA Cimica V Mackow ER. Dengue virus ns proteins inhibit RIG-I/MAVS signaling by blocking TBK1/IRF3 phosphorylation: dengue virus serotype 1 NS4A is a unique interferon-regulating virulence determinant. mBio (2015) 6:e00553–15. 10.1128/mBio.00553-15 25968648 PMC4436066

[7] Munoz-Jordan JL Sanchez-Burgos GG Laurent-Rolle M Garcia-Sastre A. Inhibition of interferon signaling by dengue virus. Pro Nat Acad Sci USA. (2003) 100:14333–8. 10.1073/pnas.2335168100 14612562 PMC283592

[8] Aguirre S Luthra P Sanchez-Aparicio MT Maestre AM Patel J Lamothe F. Dengue virus NS2B protein targets cGAS for degradation and prevents mitochondrial DNA sensing during infection. Nat Microbiol. (2017) 2:17037. 10.1038/nmicrobiol.2017.37 28346446 PMC7457382

[9] Aguirre S Maestre AM Pagni S Patel JR Savage T Gutman D. DENV inhibits type I IFN production in infected cells by cleaving human STING. PLoS Pathog. (2012) 8:e1002934. 10.1371/journal.ppat.1002934 23055924 PMC3464218

[10] Yu CY Chang TH Liang JJ Chiang RL Lee YL Liao CL. Dengue virus targets the adaptor protein MITA to subvert host innate immunity. PLoS Pathog. (2012) 8:e1002780. 10.1371/journal.ppat.1002780 22761576 PMC3386177

[11] Anglero-Rodriguez YI Pantoja P Sariol CA. Dengue virus subverts the interferon induction pathway via NS2B/3 protease-IkappaB kinase epsilon interaction. Clin Vaccine Immunol. (2014) 21:29–38. 10.1128/CVI.00500-13 24173023 PMC3910921

[12] Yu CY Liang JJ Li JK Lee YL Chang BL Su CI. Dengue virus impairs mitochondrial fusion by cleaving mitofusins. PLoS Pathog. (2015) 11:e1005350. 10.1371/journal.ppat.1005350 26717518 PMC4696832

[13] Chan YK Gack MU. A phosphomimetic-based mechanism of dengue virus to antagonize innate immunity. Nat Immunol. (2016) 17:523–30. 10.1038/ni.3393 26998762 PMC4837045

[14] He Z Zhu X Wen W Yuan J Hu Y Chen J. Dengue virus subverts host innate immunity by targeting adaptor protein MAVS. J Virol. (2016) 90:7219–30. 10.1128/JVI.00221-16 27252539 PMC4984625

[15] Chang DC Hoang LT Mohamed Naim AN Dong H Schreiber MJ Hibberd ML. Evasion of early innate immune response by 2'-O-methylation of dengue genomic RNA. Virology (2016) 499:259–66. 10.1016/j.virol.2016.09.022 27716465 PMC7172056

[16] Ashour J Laurent-Rolle M Shi PY Garcia-Sastre A. NS5 of dengue virus mediates STAT2 binding and degradation. J Virol. (2009) 83:5408–18. 10.1128/JVI.02188-08 19279106 PMC2681973

[17] Mazzon M Jones M Davidson A Chain B Jacobs M. Dengue virus NS5 inhibits interferon-alpha signaling by blocking signal transducer and activator of transcription 2 phosphorylation. J Infect. Dis. (2009) 200:1261–70. 10.1086/605847 19754307

[18] Morrison J Laurent-Rolle M Maestre AM Rajsbaum R Pisanelli G Simon V. Dengue virus co-opts UBR4 to degrade STAT2 and antagonize type I interferon signaling. PLoS Pathog. (2013) 9:e1003265. 10.1371/journal.ppat.1003265 23555265 PMC3610674

[19] Apte-Sengupta S Sirohi D Kuhn RJ. Coupling of replication and assembly in flaviviruses. Curr Opin Virol. (2014) 9:134–42. 10.1016/j.coviro.2014.09.020 25462445 PMC4268268

[20] Guzman MG Harris E. Dengue. Lancet (2015) 385:453–65. 10.1016/S0140-6736(14)60572-9 25230594

[21] Nguyet MN Duong TH Trung VT Nguyen TH Tran CN Long VT. Host and viral features of human dengue cases shape the population of infected and infectious Aedes aegypti mosquitoes. Proc Nat Acad Sci USA. (2013) 110:9072–7. 10.1073/pnas.1303395110 23674683 PMC3670336

[22] Carrington LB Simmons CP. Human to mosquito transmission of dengue viruses. Front Immunol. (2014) 5:290. 10.3389/fimmu.2014.00290 24987394 PMC4060056

[23] Conway MJ Londono-Renteria B Troupin A Watson AM Klimstra WB Fikrig E. Aedes aegypti D7 saliva protein inhibits dengue virus infection. PLoS Negl Trop Dis. (2016) 10:e0004941. 10.1371/journal.pntd.0004941 27632170 PMC5025043

[24] Conway MJ Watson AM Colpitts TM Dragovic SM Li Z Wang P. Mosquito saliva serine protease enhances dissemination of dengue virus into the mammalian host. J Virol. (2014) 88:164–75. 10.1128/JVI.02235-13 24131723 PMC3911723

[25] Schmid MA Glasner DR Shah S Michlmayr D Kramer LD Harris E. Mosquito saliva increases endothelial permeability in the skin, immune cell migration, and dengue pathogenesis during antibody-dependent enhancement. PLoS Pathog. (2016) 12:e1005676. 10.1371/journal.ppat.1005676 27310141 PMC4911004

[26] Chang TH Liao CL Lin YL. Flavivirus induces interferon-beta gene expression through a pathway involving RIG-I-dependent IRF-3 and PI3K-dependent NF-kappaB activation. Microbes Infect. (2006) 8:157–71. 10.1016/j.micinf.2005.06.014 16182584

[27] Rodriguez-Madoz JR Bernal-Rubio D Kaminski D Boyd K Fernandez-Sesma A. Dengue virus inhibits the production of type I interferon in primary human dendritic cells. J Virol. (2010) 84:4845–50. 10.1128/JVI.02514-09 20164230 PMC2863727

[28] Castillo Ramirez JA Urcuqui-Inchima S. Dengue virus control of type I IFN responses: a history of manipulation and control. J Interferon Cytokine Res. (2015) 35:421–30. 10.1089/jir.2014.0129 25629430 PMC4490770

[29] Takeuchi O Akira S. Pattern recognition receptors and inflammation. Cell (2010) 140:805–20. 10.1016/j.cell.2010.01.022 20303872

[30] Jensen S Thomsen AR. Sensing of RNA viruses: a review of innate immune receptors involved in recognizing RNA virus invasion. J Virol. (2012) 86:2900–10. 10.1128/JVI.05738-11 22258243 PMC3302314

[31] Doly J Civas A Navarro S Uze G. Type I interferons: expression and signalization. Cell Mol Life sci. (1998) 54:1109–21. 10.1007/s000180050240 9817990 PMC11147243

[32] Haller O Kochs G Weber F. The interferon response circuit: induction and suppression by pathogenic viruses. Virology (2006) 344:119–30. 10.1016/j.virol.2005.09.024 16364743 PMC7125643

[33] Lin RJ Yu HP Chang BL Tang WC Liao CL Lin YL. Distinct antiviral roles for human 2',5'-oligoadenylate synthetase family members against dengue virus infection. J Immunol. (2009) 183:8035–43. 10.4049/jimmunol.0902728 19923450

[34] Furuichi Y LaFiandra A Shatkin AJ. 5'-Terminal structure and mRNA stability. Nature (1977) 266:235–9. 557727 10.1038/266235a0

[35] Hyde JL Diamond MS Innate immune restriction and antagonism of viral RNA lacking 2-O methylation. Virology (2015) 479–480:66–74. 10.1016/j.virol.2015.01.019 PMC4424151 25682435

[36] Daffis S Szretter KJ Schriewer J Li J Youn S Errett J. 2'-O methylation of the viral mRNA cap evades host restriction by IFIT family members. Nature (2010) 468:452–6. 10.1038/nature09489 21085181 PMC3058805

[37] Zust R Cervantes-Barragan L Habjan M Maier R Neuman BW Ziebuhr J. Ribose 2'-O-methylation provides a molecular signature for the distinction of self and non-self mRNA dependent on the RNA sensor Mda5. Nat Immunol. (2011) 12:137–43. 10.1038/ni.1979 21217758 PMC3182538

[38] Hyde JL Gardner CL Kimura T White JP Liu G Trobaugh DW. A viral RNA structural element alters host recognition of nonself RNA. Science (2014) 343:783–7. 10.1126/science.1248465 24482115 PMC4209899

[39] Egloff MP Decroly E Malet H Selisko B Benarroch D Ferron F. Structural and functional analysis of methylation and 5'-RNA sequence requirements of short capped RNAs by the methyltransferase domain of dengue virus NS5. J Mol Biol. (2007) 372:723–36. 10.1016/j.jmb.2007.07.005 17686489

[40] Liu L Dong H Chen H Zhang J Ling H Li Z. Flavivirus RNA cap methyltransferase: structure, function, and inhibition. Front Biol. (2010) 5:286–303. 10.1007/s11515-010-0660-y 21927615 PMC3172701

[41] Dong H Fink K Zust R Lim SP Qin CF Shi PY. Flavivirus RNA methylation. J Gen Virol. (2014) 95(Pt 4):763–78. 10.1099/vir.0.062208-0 24486628

[42] Welsch S Miller S Romero-Brey I Merz A Bleck CK Walther P. Composition and three-dimensional architecture of the dengue virus replication and assembly sites. Cell Host Microbe (2009) 5:365–75. 10.1016/j.chom.2009.03.007 19380115 PMC7103389

[43] Uchida L Espada-Murao LA Takamatsu Y Okamoto K Hayasaka D Yu F. The dengue virus conceals double-stranded RNA in the intracellular membrane to escape from an interferon response. Sci Rep. (2014) 4:7395. 10.1038/srep07395 25491663 PMC4261170

[44] Nasirudeen AM Wong HH Thien P Xu S Lam KP Liu DX. RIG-I, MDA5 and TLR3 synergistically play an important role in restriction of dengue virus infection. PLoS Negl Trop Dis. (2011) 5:e926. 10.1371/journal.pntd.0000926 21245912 PMC3014945

[45] Surasombatpattana P Hamel R Patramool S Luplertlop N Thomas F Despres P. Dengue virus replication in infected human keratinocytes leads to activation of antiviral innate immune responses. Infect Genet Evol. (2011) 11:1664–73. 10.1016/j.meegid.2011.06.009 21722754

[46] Urcuqui-Inchima S Cabrera J Haenni AL. Interplay between dengue virus and Toll-like receptors, RIG-I/MDA5 and microRNAs: implications for pathogenesis. Antiviral Res. (2017) 147:47–57. 10.1016/j.antiviral.2017.09.017 28965915

[47] Meylan E Curran J Hofmann K Moradpour D Binder M Bartenschlager R. Cardif is an adaptor protein in the RIG-I antiviral pathway and is targeted by hepatitis C virus. Nature (2005) 437:1167–72. 10.1038/nature04193 16177806

[48] Kawai T Takahashi K Sato S Coban C Kumar H Kato H. IPS-1, an adaptor triggering RIG-I- and Mda5-mediated type I interferon induction. Nat. Immunol. (2005) 6:981–8. 10.1038/ni1243 16127453

[49] Seth RB Sun L Ea CK Chen ZJ. Identification and characterization of MAVS, a mitochondrial antiviral signaling protein that activates NF-kappaB and IRF 3. Cell (2005) 122:669–82. 10.1016/j.cell.2005.08.012 16125763

[50] Xu LG Wang YY Han KJ Li LY Zhai Z Shu HB. VISA is an adapter protein required for virus-triggered IFN-beta signaling. Mol Cell (2005) 19:727–40. 10.1016/j.molcel.2005.08.014 16153868

[51] Kato H Takeuchi O Mikamo-Satoh E Hirai R Kawai T Matsushita K. Length-dependent recognition of double-stranded ribonucleic acids by retinoic acid-inducible gene-I and melanoma differentiation-associated gene 5. J Exp Med. (2008) 205:1601–10. 10.1084/jem.20080091 18591409 PMC2442638

[52] Takahasi K Yoneyama M Nishihori T Hirai R Kumeta H Narita R. Nonself RNA-sensing mechanism of RIG-I helicase and activation of antiviral immune responses. Mol Cell (2008) 29:428–40. 10.1016/j.molcel.2007.11.028 18242112

[53] Weber M Gawanbacht A Habjan M Rang A Borner C Schmidt AM. Incoming RNA virus nucleocapsids containing a 5'-triphosphorylated genome activate RIG-I and antiviral signaling. Cell Host Microbe (2013) 13:336–46. 10.1016/j.chom.2013.01.012 23498958 PMC5515363

[54] Loo YM Fornek J Crochet N Bajwa G Perwitasari O Martinez-Sobrido L. Distinct RIG-I and MDA5 signaling by RNA viruses in innate immunity. J Virol. (2008) 82:335–45. 10.1128/JVI.01080-07 17942531 PMC2224404

[55] Yu CY Chiang RL Chang TH Liao CL Lin YL. The interferon stimulator mitochondrial antiviral signaling protein facilitates cell death by disrupting the mitochondrial membrane potential and by activating caspases. J Virol. (2010) 84:2421–31. 10.1128/JVI.02174-09 20032188 PMC2820939

[56] Perry ST Prestwood TR Lada SM Benedict CA Shresta S. Cardif-mediated signaling controls the initial innate response to dengue virus *in vivo*. J Virol. (2009) 83:8276–81. 10.1128/JVI.00365-09 19494017 PMC2715757

[57] Schoggins JW Rice CM. Interferon-stimulated genes and their antiviral effector functions. Curr Opin Virol. (2011) 1:519–25. 10.1016/j.coviro.2011.10.008 22328912 PMC3274382

[58] Gack MU Shin YC Joo CH Urano T Liang C Sun L. TRIM25 RING-finger E3 ubiquitin ligase is essential for RIG-I-mediated antiviral activity. Nature (2007) 446:916–20. 10.1038/nature05732 17392790

[59] Gack MU Kirchhofer A Shin YC Inn KS Liang C Cui S. Roles of RIG-I N-terminal tandem CARD and splice variant in TRIM25-mediated antiviral signal transduction. Proc Nat Acad Sci USA. (2008) 105:16743–8. 10.1073/pnas.0804947105 18948594 PMC2575490

[60] Peisley A Wu B Xu H Chen ZJ Hur S. Structural basis for ubiquitin-mediated antiviral signal activation by RIG-I. Nature (2014) 509:110–4. 10.1038/nature13140 24590070 PMC6136653

[61] Liu HM Loo YM Horner SM Zornetzer GA Katze MG Gale M Jr. The mitochondrial targeting chaperone 14-3-3epsilon regulates a RIG-I translocon that mediates membrane association and innate antiviral immunity. Cell Host Microbe (2012) 11:528–37. 10.1016/j.chom.2012.04.006 22607805 PMC3358705

[62] Pijlman GP Funk A Kondratieva N Leung J Torres S van der Aa L. A highly structured, nuclease-resistant, noncoding RNA produced by flaviviruses is required for pathogenicity. Cell Host Microbe (2008) 4:579–91. 10.1016/j.chom.2008.10.007 19064258

[63] Heil F Hemmi H Hochrein H Ampenberger F Kirschning C Akira S. Species-specific recognition of single-stranded RNA via toll-like receptor 7 and 8. Science (2004) 303:1526–9. 10.1126/science.1093620 14976262

[64] Tsai YT Chang SY Lee CN Kao CL. Human TLR3 recognizes dengue virus and modulates viral replication *in vitro*. Cel Microbiol. (2009) 11:604–15. 10.1111/j.1462-5822.2008.01277.x 19134117

[65] Sariol CA Martinez MI Rivera F Rodriguez IV Pantoja P Abel K. Decreased dengue replication and an increased anti-viral humoral response with the use of combined Toll-like receptor 3 and 7/8 agonists in macaques. PLoS ONE (2011) 6:e19323. 10.1371/journal.pone.0019323 21559444 PMC3084804

[66] Sparrer KM Gack MU. Intracellular detection of viral nucleic acids. Curr Opin Microbiol. (2015) 26:1–9. 10.1016/j.mib.2015.03.001 25795286 PMC5084527

[67] Sun L Wu J Du F Chen X Chen ZJ. Cyclic GMP-AMP synthase is a cytosolic DNA sensor that activates the type I interferon pathway. Science (2013) 339:786–91. 10.1126/science.1232458 23258413 PMC3863629

[68] Chen Q Sun L Chen ZJ. Regulation and function of the cGAS-STING pathway of cytosolic DNA sensing. Nat Immunol. (2016) 17:1142–9. 10.1038/ni.3558 27648547

[69] Ishikawa H Barber GN. STING is an endoplasmic reticulum adaptor that facilitates innate immune signalling. Nature (2008) 455:674–8. 10.1038/nature07317 18724357 PMC2804933

[70] Zhong B Yang Y Li S Wang YY Li Y Diao F. The adaptor protein MITA links virus-sensing receptors to IRF3 transcription factor activation. Immunity (2008) 29:538–50. 10.1016/j.immuni.2008.09.003 18818105

[71] Sun W Li Y Chen L Chen H You F Zhou X. ERIS, an endoplasmic reticulum IFN stimulator, activates innate immune signaling through dimerization. Proc Nat Acad Sci USA. (2009) 106:8653–8. 10.1073/pnas.0900850106 19433799 PMC2689030

[72] Jin L Waterman PM Jonscher KR Short CM Reisdorph NA Cambier JC. MPYS, a novel membrane tetraspanner, is associated with major histocompatibility complex class II and mediates transduction of apoptotic signals. Mol Cell Biol. (2008) 28:5014–26. 10.1128/MCB.00640-08 18559423 PMC2519703

[73] Abe T Barber GN. Cytosolic-DNA-mediated, STING-dependent proinflammatory gene induction necessitates canonical NF-kappaB activation through TBK1. J Virol. (2014) 88:5328–41. 10.1128/JVI.00037-14 24600004 PMC4019140

[74] Li Y Wilson HL Kiss-Toth E. Regulating STING in health and disease. J Inflammation (2017) 14:11. 10.1186/s12950-017-0159-2 28596706 PMC5463399

[75] Zevini A Olagnier D Hiscott J. Crosstalk between Cytoplasmic RIG-I and STING Sensing Pathways. Trends Immunol. (2017) 38:194–205. 10.1016/j.it.2016.12.004 28073693 PMC5329138

[76] Rodriguez-Madoz JR Belicha-Villanueva A Bernal-Rubio D Ashour J Ayllon J Fernandez-Sesma A. Inhibition of the type I interferon response in human dendritic cells by dengue virus infection requires a catalytically active NS2B3 complex. J Virol. (2010) 84:9760–74. 10.1128/JVI.01051-10 20660196 PMC2937777

[77] Platanias LC. Mechanisms of type-I- and type-II-interferon-mediated signalling. Nat Rev Immunol. (2005) 5:375–86. 10.1038/nri1604 15864272

[78] Ivashkiv LB Donlin LT. Regulation of type I interferon responses. Nat Rev Immunol. (2014) 14:36–49. 10.1038/nri3581 24362405 PMC4084561

[79] Kim TK Maniatis T. Regulation of interferon-gamma-activated STAT1 by the ubiquitin-proteasome pathway. Science (1996) 273:1717–9. 8781235 10.1126/science.273.5282.1717

[80] Yeh HM Yu CY Yang HC Ko SH Liao CL Lin YL. Ubiquitin-specific protease 13 regulates IFN signaling by stabilizing STAT1. J Immunol. (2013) 191:3328–36. 10.4049/jimmunol.1300225 23940278

[81] Su CI Tseng CH Yu CY Lai MMC. SUMO modification stabilizes dengue virus nonstructural protein 5 to support virus replication. J. Virol. (2016) 90:4308–19. 10.1128/JVI.00223-16 26889037 PMC4836324

[82] Palmer CS Osellame LD Stojanovski D Ryan MT. The regulation of mitochondrial morphology: intricate mechanisms and dynamic machinery. Cell Signal. (2011) 23:1534–45. 10.1016/j.cellsig.2011.05.021 21683788

[83] Youle RJ van der Bliek AM. Mitochondrial fission, fusion, and stress. Science (2012) 337:1062–5. 10.1126/science.1219855 22936770 PMC4762028

[84] Hou F Sun L Zheng H Skaug B Jiang QX Chen ZJ. MAVS forms functional prion-like aggregates to activate and propagate antiviral innate immune response. Cell (2011) 146:448–61. 10.1016/j.cell.2011.06.041 21782231 PMC3179916

[85] Onoguchi K Onomoto K Takamatsu S Jogi M Takemura A Morimoto S. Virus-infection or 5'ppp-RNA activates antiviral signal through redistribution of IPS-1 mediated by MFN1. PLoS Pathog. (2010) 6:e1001012. 10.1371/journal.ppat.1001012 20661427 PMC2908619

[86] Koshiba T Yasukawa K Yanagi Y Kawabata S. Mitochondrial membrane potential is required for MAVS-mediated antiviral signaling. Sci. Signal. (2011) 4:ra7. 10.1126/scisignal.2001147 21285412

[87] Ishihara N Jofuku A Eura Y Mihara K. Regulation of mitochondrial morphology by membrane potential, and DRP1-dependent division and FZO1-dependent fusion reaction in mammalian cells. Biochem Biophys Res Commun. (2003) 301:891–8. 10.1016/S0006-291X(03)00050-0 12589796

[88] Chatel-Chaix L Cortese M Romero-Brey I Bender S Neufeldt CJ Fischl W. Dengue virus perturbs mitochondrial morphodynamics to dampen innate immune responses. Cell Host Microbe (2016) 20:342–56. 10.1016/j.chom.2016.07.008 27545046 PMC7105029

